# Influence of Biological Sex on Metabolic and Adipokine Responses after Metabolic-Bariatric Surgery: A Narrative Review

**DOI:** 10.1007/s13679-026-00740-5

**Published:** 2026-07-22

**Authors:** Gonzalo Rivero, Amaia Rodríguez, Victoria Catalán, Beatriz Ramírez, Federico Carbone, Fabrizio Montecucco, Javier Gómez-Ambrosi, Gema Frühbeck, Sara Becerril

**Affiliations:** 1 Metabolic Research Laboratory, Clínica Universidad de Navarra, Pamplona, Spain; 2 Department of Endocrinology, Hospital Universitario Puerta de Hierro, Madrid, Majadahonda Spain; 3 CIBER Fisiopatología de la Obesidad y Nutrición (CIBEROBN), Instituto de Salud Carlos III, Madrid, Spain; 4 Obesity and Adipobiology Group, Instituto de Investigación Sanitaria de Navarra, IdiSNA, Pamplona, Spain; 5 Institute for Nutrition & Health, Clínica Universidad de Navarra, Pamplona, Spain; 6 First Clinic of Internal Medicine, Department of Internal Medicine, University of Genoa, Genoa, Italy; 7 IRCCS Ospedale Policlinico San Martino Genoa - Italian Cardiovascular Network, Genoa, Italy; 8 Department of Endocrinology & Nutrition, Clínica Universidad de Navarra, Pamplona, Spain

**Keywords:** Sexual dimorphism, Bariatric surgery, Sex differences, Obesity

## Abstract

**Purpose of review:**

Obesity is a multifactorial, chronic, and relapsing non-communicable disease characterized by excessive adipose tissue accumulation and metabolic dysfunction. Increasing evidence indicates that biological sex influences not only the development and distribution of adipose tissue but also responses to obesity treatments, including metabolic-bariatric surgery. This narrative review synthesizes current evidence on sex-related differences in metabolic outcomes after bariatric surgery.

**Recent findings:**

Bariatric surgery induces substantial weight loss and marked improvement of obesity-related complications in both men and women along sex-specific differences in body composition changes, adipose tissue remodeling, adipokine secretion, inflammatory responses, and thermogenic adaptations. Estrogen signaling contributes to a more favorable adipose tissue phenotype through anti-inflammatory effects, enhanced adipokine profiles, preservation of lean mass, and stimulation of white adipose tissue beiging, a process by which an inducible form of thermogenic adipocytes within white adipose tissue (beige adipocytes) acquire thermogenic characteristics similar to brown adipose tissue. In women, menopausal status represents a key biological modifier of these responses. In contrast, androgen excess is associated with visceral fat accumulation and insulin resistance. Despite largely comparable rates of remission of major obesity-related complications between sexes, variances in adiposity, hormonal milieu, and postoperative metabolic outcomes suggest different underlying mechanisms.

**Summary:**

Although remission rates of obesity-related complications appear broadly comparable between sexes, the underlying metabolic and endocrine adaptations differ. Biological sex represents a determinant of post-bariatric metabolic remodeling. This review highlights the relevance of biological sex in obesity and its treatment, emphasizing the need for sex-stratified studies to elucidate underlying mechanisms and support the development of more precise, personalized strategies in obesity management.

## Key Messages

Biological sex influences metabolic and compositional outcomes following bariatric surgery, including adipose tissue remodeling, adipokine secretion, and body composition changes.

The direction and magnitude of reported sex differences are not fully consistent across studies.

Apparent sex-related differences are frequently shaped by baseline disparities in age, BMI, adiposity, and complications.

Menopause critically modifies adipose tissue distribution, inflammatory status, and thermogenic activity, and should be systematically considered in future studies.

Procedure selection (e.g., SG vs. RYGB) and heterogeneity in follow-up duration further limit direct comparisons.

## Introduction

Obesity is considered a multifactorial, chronic, relapsing, non-communicable disease characterized by an excessive accumulation of adipose tissue [1]. This altered adipose tissue accumulation is closely associated with a chronic low-grade proinflammatory state, which adversely affects overall health [2]. This adverse health impact manifests in conditions like type 2 diabetes, hypertension or dyslipidemia. The underlying pathological mechanisms ultimately contribute to the development of severe complications including myocardial infarction, stroke, atherosclerosis, and several types of cancers that can compromise both life expectancy and quality of life [3, 4].

Despite the considerable social and economic burden of obesity, the impact of biological sex has only recently begun to receive attention. Sexual dimorphisms affect multiple aspects of obesity, ranging from disease mechanisms to clinical outcomes of both pharmacological and surgical interventions [5]. The marked sexual differences in adipose tissue biology, hormone signaling and inflammatory responses may critically influence both the short- and long-term outcomes of bariatric surgery. Therefore, further research into sex-specific factors is essential to advance personalized medicine, optimizing potential benefits while reducing adverse effects. In line with current terminology proposed by the International Federation for the Surgery of Obesity and Metabolic Disorders (IFSO), the term “metabolic-bariatric surgery” is increasingly preferred [6], reflecting the broad metabolic and endocrine benefits of these procedures beyond weight loss alone.

## Methodology

A comprehensive and structured literature search was performed using major biomedical databases, including PubMed, Embase, and Cochrane Library, employing the following Medical Subject Headings (MeSH) terms: “sexual dimorphism” combined with “bariatric surgery”, “biological sex”, “adipokines”, “obesity”, “obesity treatment”, “obesity management”, “gastric bypass” and “biliopancreatic diversion”. The literature search was conducted between January and March 2025. Titles and abstracts were screened independently by two authors, and potentially relevant studies were assessed in full text according to their relevance to the thematic scope of the review. Boolean operators (AND/OR) were used to refine the search strategy and ensure relevance. This search strategy was designed to support a narrative synthesis of the available literature regarding biological sex-related differences in obesity and metabolic adaptations following bariatric surgery. Only full-text articles published in English were included. Duplicates, irrelevant articles, case reports, and letters were excluded. Both clinical and preclinical studies were included when considered relevant to the mechanistic interpretation of sex-specific responses. Articles were selected based on their relevance to the thematic focus of the review. The retrieved literature was organized into thematic sections based on the main biological and clinical areas related to obesity and bariatric surgery outcomes.

## Epidemiology and Definition of Obesity

### Global and Sex-specific Prevalence

The prevalence of obesity has shown a progressive increase in recent decades, affecting both sexes across all age groups and representing an important global public health challenge. In 2021, excess body weight was estimated to be responsible for 3.71 million deaths and 129 million disability-adjusted life years [7]. Moreover, projections suggest that the global economic burden will reach USD 4 trillion by 2035 [8]. These alarming trends underscore the critical need for a deeper understanding not only of the pathophysiology of obesity and the identification of novel therapeutic targets, but also for supporting sustainable economic growth.

Sex-stratified analyses reveal notable differences in obesity prevalence. Globally, approximately 11% of men and 15% of women are classified as having obesity [9]. This disparity is evident across many regions of the world and likely reflects a combination of biological, sociocultural and behavioral factors that contribute to sex-specific disparities in obesity prevalence [10].

### Diagnosis of Obesity and Body Mass Index Limitations

Obesity has traditionally been defined as a body mass index (BMI) equal to or greater than 30 kg/m² [11]. However, the use of BMI as the sole diagnostic criterion has increasingly been questioned due to its inherent limitations. This anthropometric approach does not capture the heterogeneity of obesity, including its diverse phenotypes and the variable distribution and function of adipose tissue [12–14]. Studies conducted in individuals with BMI values within the overweight (≥ 25 and ≤ 30 kg/m²) or normal ranges (18.5–24.9 kg/m²), assessed using gold-standard body composition methods such as air displacement plethysmography, exhibited elevated visceral adiposity and increased cardiometabolic risk [13]. Recent expert consensus statements recommend contemplating obesity as an adiposity-based chronic disease, encouraging clinicians to incorporate complementary metrics that better reflect obesity-related health risks [15]. Among these, the waist-to-height ratio shares this simplicity but better correlates with the metabolic syndrome [16].

## Adipose Tissue and Biological Sex Dimorphism

### Classification of Adipose Tissue

Adipose tissue is a dynamic organ with distinct anatomical and functional depots, each contributing to energy balance and systemic metabolism [17]. Obesity is characterized by an abnormal and excessive accumulation of adipose tissue in different anatomical depots. Adipose tissue can be classified according to both its anatomical distribution as well as its physiological characteristics. Subcutaneous adipose tissue, located beneath the dermis, constitutes the primary energy reservoir [18]. Subcutaneous adipose tissue exhibits lower basal lipolytic activity, greater insulin sensitivity, and a relatively anti-inflammatory secretory profile, features that collectively confer a more favorable cardiometabolic profile compared with the visceral depot [19]. Visceral adipose tissue is predominantly accumulated intra-abdominally, surrounding internal organs. Visceral adiposity is characterized by elevated lipolytic activity [20, 21], insulin resistance, and a proinflammatory state [22], being strongly associated with adverse metabolic dysfunction [23].

Brown adipose tissue is specialized in non-shivering thermogenesis and plays an important role in energy expenditure and temperature regulation [24]. Under certain stimuli, such as exercise, cold exposure, or β-adrenergic receptor activation, white adipocytes can acquire a more thermogenic profile. This process is referred to as “beiging” or “browning” [25].

Beige adipose tissue differs from mature brown adipose tissue and white adipose tissue by exhibiting distinct cellular characteristics, including multilocular lipid droplets, a high mitochondrial content, and an enhanced thermogenic capacity mediated by uncoupling protein 1 (UCP1) together with other thermogenic factors such as T-box transcription factor 1 (TBX1), transmembrane protein 26 (TMEM26) or CD137 [26]. This adaptive plasticity and the capacity to promote thermogenesis, increasing basal metabolic rate, highlights the potential of beige fat activation as a therapeutic target for the treatment of obesity [27].

### Sexual Dimorphism in Adipose Tissue Distribution and Expansion

**Fig. 1:**
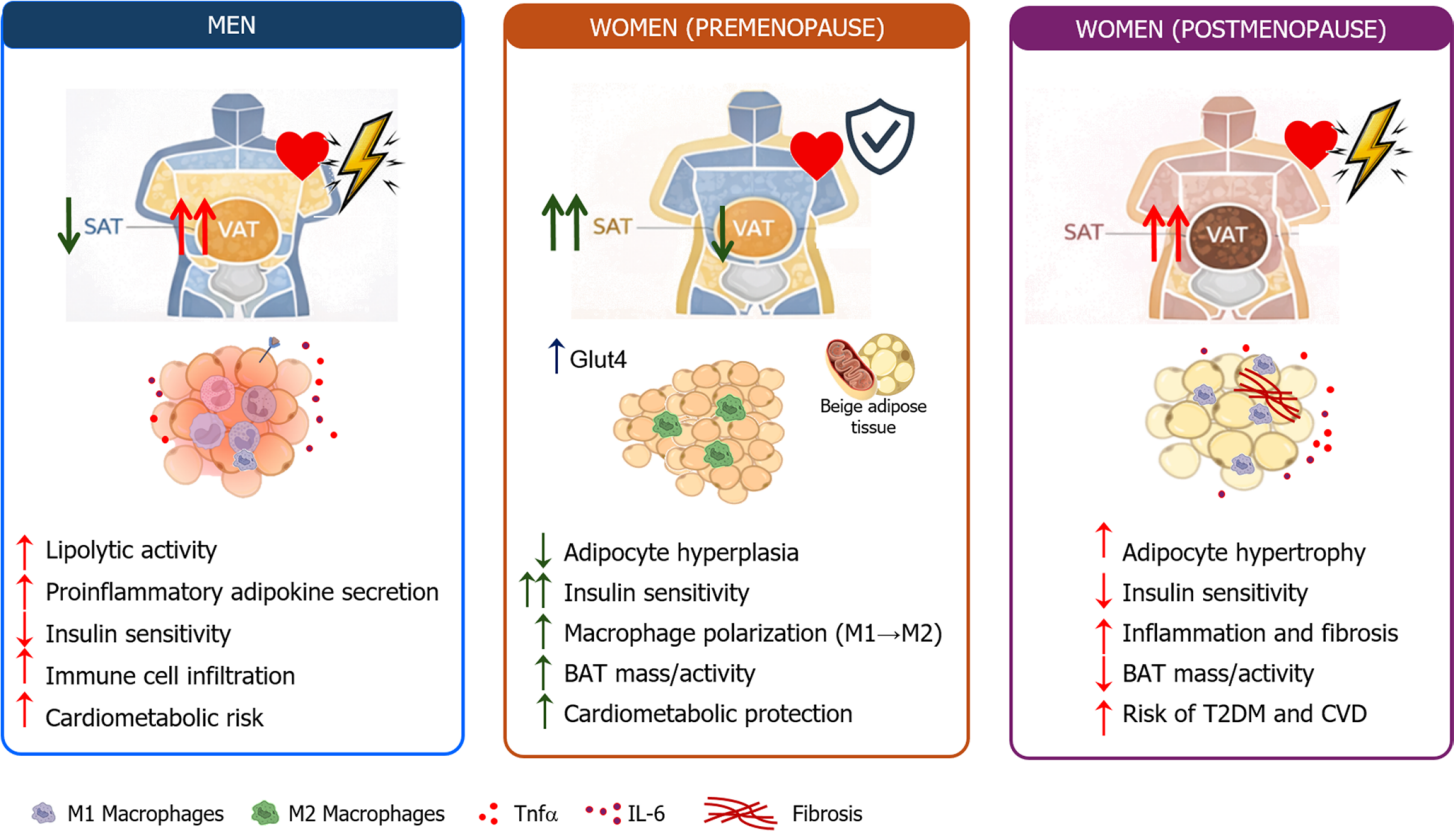
Sex-dependent differences in adipose tissue distribution and metabolic profiles. Men typically exhibit higher visceral adipose tissue accumulation associated with increased lipolytic activity, elevated pro-inflammatory adipokine secretion (TNF-α, IL-6), and increased cardiometabolic risk. In contrast, premenopausal women predominantly accumulate subcutaneous adipose tissue, higher insulin sensitivity, and enhanced brown adipose tissue activity, collectively conferring cardiometabolic protection. However, following menopause, women experience a shift toward a more male-like adipose phenotype, with increased visceral adipose tissue deposition, adipocyte hypertrophy, and a heightened risk for type 2 diabetes and cardiovascular disease, concomitant with the decline in estrogen-mediated protection. Abbreviations: BAT, brown adipose tissue, CDV, cardiovascular disease; SAT, subcutaneous adipose tissue; T2DM, type 2 diabetes; VAT, visceral adipose tissue

Regarding the functional characteristics of adipose depots described above, sexual dimorphism significantly influences the distribution and expansion of adipose tissue. Quantitative studies reveal that men predominantly accumulate adipose tissue within visceral depots, whereas women primarily store adipose tissue in subcutaneous depots [28]. However, postmenopausal women typically experience a shift in body fat distribution from the gluteo-femoral region to the abdomen and trunk (29). The sex-specific distribution contributes to differential cardiometabolic risk, with men being more prone to visceral fat-associated insulin resistance and inflammation [30]. Additionally, the proportion of brown adipose tissue in females is higher than in males, potentially contributing to their relatively higher basal metabolic rate and enhanced thermogenesis [31, 32] (Fig. 1).

Evidence from animal models indicates that estrogens promote adipocyte differentiation and adipogenesis as well as the gene expression pathways that regulate insulin sensitivity and lipogenesis [33]. Furthermore, estrogens also influence an inflammatory environment and adipose tissue remodeling responses, processes that are pivotal in both the development and progression of obesity, at the same time as in determining outcomes following bariatric surgery [34]. Conversely, androgen excess favors tissue inflammation and insulin resistance, whereas androgen-targeted therapies can partially reverse these adverse fat distribution patterns [35].

### Influence of Sex Hormones on Adipose Tissue

Sex-specific differences in adipose tissue distribution are largely mediated by estrogens and manifest over the female life course, including puberty and menopause, as well as in conditions characterized by altered sex hormone levels, such as polycystic ovary syndrome and in transgender individuals undergoing gender-affirming hormone therapy [36]. Estrogens regulate adipose tissue deposition through their receptors, estrogen receptor alpha (ERα) and estrogen receptor beta (ERβ), which are expressed in both visceral and subcutaneous adipose tissue [33]. This has also been demonstrated in experimental animal models [37].

Evidence indicates that male mice exhibit lower expression of ERα and ERβ proteins in white adipose tissue compared with females. Furthermore, *Era* deficiency in mice results in adipocyte hyperplasia, insulin resistance, glucose intolerance, and hepatic steatosis regardless of sex [38]. This adipocyte hyperplasia has been linked to the role of estrogens in inhibiting peroxisome proliferator-activated receptor γ (PPARγ), a key regulator of adipogenesis, leading to the downregulation of genes involved in lipid storage and adipocyte differentiation [39]. Consequent alterations in adipose tissue, including excess circulating free fatty acids and lipid accumulation disrupt cellular metabolism, leading to mitochondrial dysfunction, endoplasmic reticulum stress, hypoxia, cell death, and fibrosis [40]. These alterations promote macrophage recruitment and activation, triggering the secretion of proinflammatory cytokines such as tumor necrosis factor-alpha (TNF-α) and interleukin 6 (IL-6). Estrogens modulate this immune response, as observed in *Era/b* knockout mice, which exhibit increased adipose tissue inflammation evidenced by the upregulation of *Il6* and *Tnf* gene expression, underscoring the anti-inflammatory role of estrogen-signaling. Collectively, these findings highlight the crucial role of ERα in maintaining normal adipose tissue expansion and metabolic homeostasis.

Clinical studies in transgender people highlight the contribution of sex hormones in adipose tissue distribution. Tebbens et al. [41] reported that trans women (assigned male at birth) undergoing anti-androgen therapy exhibited a shift within adipose tissue distribution, with an increase in subcutaneous fat and a reduction in visceral adipose tissue. Conversely, individuals transitioning to male and initiating testosterone therapy exhibited a predominant visceral fat accumulation. These observations underscore the concept that sex hormones, especially estrogens and androgens, are key determinants of fat distribution. Supporting this notion, the progressive decline in estrogen levels following menopause is associated with a redistribution of excess adiposity toward the visceral depot and a concomitant deterioration of the cardiometabolic profile, independently of chronological age [42].

Beyond regulating fat distribution, estrogens enhance thermogenesis through central, peripheral, and immunometabolic pathways, promoting beiging of white adipose tissue via ERα. This process increases sympathetic tone in brown adipose tissue and activates UCP1 independently of food intake [43]. Collectively, these sex-dependent processes of adipose expansion, immune regulation, and metabolic signaling likely influence postoperative improvements in insulin sensitivity, lipid metabolism, and energy balance. Understanding these estrogen-derived mechanisms is, therefore, crucial for interpreting sex-specific responses to bariatric surgery.

### Adipokines and Sex Differences

Adipose tissue functions as a secretory organ involved in systemic metabolic regulation. Beyond acting as an energy reservoir, adipose tissue is involved in the regulation of energy expenditure, insulin sensitivity, bone metabolism, immune and inflammatory responses as well as endocrine and reproductive functions [44]. Adipokines, cell bioactive signaling proteins secreted by adipose tissue, act as key mediators of these processes, controlling metabolic homeostasis through the regulation of carbohydrate and lipid metabolism [45–47].

Sex hormones, particularly estrogens, play an important role in the regulation of adipokine expression and activity. Therefore, in women, menopausal status may further modulate adipokine profiles, as estrogen decline is associated with increased visceral adiposity and inflammatory remodeling of adipose tissue [33]. Among adipokines, leptin is a key regulator of both weight control by regulating both caloric intake and energy expenditure [48]. Interestingly, estrogens regulate leptin expression not only via circulating estrogen levels, but also through the relative expression of ERα to ERβ in adipose tissue, highlighting a tissue-specific mechanism of action [49]. In rodent models, male rats fed a high-fat diet show a more pronounced increase in circulating leptin concentrations, accompanied by reduced hypothalamic leptin sensitivity, whereas females maintain greater leptin sensitivity and more efficient appetite regulation [50, 51]. Gonadectomy and hormone replacement experiments in rodents further support these findings: ovariectomy reduces leptin sensitivity and increases food intake, effects that can be reversed by estradiol administration. These observations collectively indicate that leptin dynamics in diet-induced obesity are sex-dependent and are tightly regulated by gonadal hormones, adipose tissue distribution, and hypothalamic sensitivity.

Adiponectin, in turn, is a crucial adipokine that exhibits anti-inflammatory, insulin-sensitizing and cardioprotective properties [52]. Circulating adiponectin levels are reduced in people living with obesity and in experimental animals fed a high-calorie diet, highlighting its inverse relationship with adiposity [53]. Notably, adiponectin demonstrates a clear sexual dimorphism, with higher circulating levels in women, a phenomenon likely mediated by estrogenic activity [54]. The adiponectin-to-leptin ratio has emerged as a robust marker of metabolic risk, being closely associated with insulin sensitivity [55–57]. This ratio tends to be higher in men, primarily due to substantially elevated baseline leptin concentrations in women [58].

Ghrelin, an orexigenic hormone, further contributes to the sex-dependent regulation of energy homeostasis [59]. Secreted mainly by the stomach, with smaller amounts produced in the pancreas, lungs, kidneys, and pituitary gland, ghrelin rises preprandially and falls in the postprandial period, highlighting its role in appetite control [60]. Despite heterogeneity among studies, a recent meta-analysis reported that fasting ghrelin levels are reduced in obesity, consistent with the hypothesis of a compensatory downregulation in the context of energy surplus [61]. Women exhibit higher preprandial ghrelin levels than men [62] but show an earlier postprandial suppression, suggesting a more rapid onset of satiety [63].

Beyond traditional adipokines, the growth hormone (GH) axis represents a critical component of metabolic regulation that exhibits significant sexual dimorphism. GH secretion is characterized by a pulsatile pattern in both sexes but women tend to exhibit higher basal secretion and more frequent pulses, largely driven by the stimulatory effects of estrogens on the pituitary [64]. In obesity, individuals experience a “functional hyposomatotropism,” where excessive accumulation of adipose tissue leads to a profound suppression of GH secretion [65]. This suppression is often more pronounced in men than in women for any given body mass index (BMI), suggesting that the negative feedback from free fatty acids and hyperinsulinemia on the GH axis is modulated by biological sex [66].

Additional adipokines also contribute to metabolic regulation. Resistin, a pro-inflammatory adipokine predominantly expressed by macrophages in humans and by adipose tissue in mice is considered a marker of low-grade inflammation, cardiovascular disease, and insulin resistance [67]. In obesity, increased resistin levels both in humans [68] and in murine models [69] have been reported, although evidence regarding sexual dimorphism is limited and well-designed studies are still needed. Visfatin, an adipokine that has been implicated in insulin resistance, is secreted mainly by visceral adipose tissue and correlates positively with BMI [70]. Visfatin does not appear to exhibit clear sexual dimorphism in circulating levels, suggesting that it is not strongly regulated by sex hormones [71].

### Sex Differences in Adipose Tissue Function

Compared with men, premenopausal women exhibit a greater capacity to remodel and expand gluteo-femoral adipose tissue through hyperplasia, promoting lipid storage in subcutaneous rather than visceral depots and thereby preserving a healthier metabolic, endocrine, and inflammatory profile [72]. Adrenergic receptor expression further shapes depot-specific lipolysis: β_1_ and β_2_ adrenergic receptors promote lipolysis, whereas α_2_-adrenergic receptors inhibit it, with receptor abundance and distribution regulating visceral fat accumulation and metabolic risk [73, 74].

Epidemiological studies indicate that males develop type 2 diabetes at lower BMI thresholds than females, reflecting differential susceptibility to insulin resistance [75]. This disparity is largely mediated by gonadal hormones, which regulate glucose homeostasis and insulin sensitivity [76] through β cell function, peripheral glucose uptake and inflammatory responses, all of which contribute to the development of insulin resistance and metabolic dysfunction [77–79]. However, this female metabolic advantage decreases during menopause, as the decline in ovarian hormone production during menopause is associated with increased visceral adiposity, impaired insulin sensitivity, and a less favorable inflammatory profile.

## Obesity Treatment

Obesity management requires a comprehensive and multidisciplinary approach, combining lifestyle modifications, pharmacological therapies, and, when appropriate, bariatric surgery [80]. Sex-related differences in obesity have significant implications for both prevention and treatment strategies, with a personalized approach to obesity management being required. Biological factors, such as hormonal profile and genetic predisposition as well as behavioral differences and psychosocial variables contribute to sex-specific variations in obesity prevalence, associated complications, and treatment responses [9, 10].

### Nonpharmacological Interventions and Sex Differences

Lifestyle interventions, including dietary modification, physical activity, and behavioral therapy, remain the cornerstone of obesity management [16]. Nevertheless, several studies indicate that men and women respond differently to lifestyle-based interventions [81]. Women generally show greater adherence to dietary programs, but lower engagement in high-intensity physical activity, while men tend to achieve greater visceral fat reductions and improvements in cardiometabolic parameters even at comparable levels of weight loss [82]. These findings highlight that these interventions should be tailored to biological sex to optimize clinical outcomes and long-term maintenance.

### Pharmacological Interventions and Sex-Based Response

Pharmacological treatment of obesity aims to achieve sustained weight loss and improve associated comorbidities by targeting metabolic pathways. These therapies are indicated for patients with obesity (BMI ≥ 30 kg/m^2^) or overweight (BMI ≥ 27 kg/m^2^) who also present obesity-related complications, such as type 2 diabetes, hypertension or dyslipidemia. Pharmacological agents exert their effects through diverse mechanisms. Glucagon-like peptide-1 (GLP-1) receptor agonists, such as semaglutide and liraglutide, and the naltrexone-bupropion combination primarily act centrally to suppress appetite [80]. GLP-1 receptor agonists additionally improve glycemic control by stimulating insulin secretion and suppressing glucagon release at the pancreatic level. Orlistat, a pancreatic lipase inhibitor, reduces dietary lipid absorption by inhibiting triglyceride hydrolysis in the gastrointestinal tract, although its weight loss efficacy is lower compared to GLP-1 receptor agonists. While pharmacological treatments for obesity are less invasive than surgical options, they generally achieve comparatively lower weight loss and are less effective in controlling obesity- associated complications in severe obesity [83].

Emerging evidence suggests that sex influences responses to GLP-1 receptor agonists, with women often achieving greater weight loss than men [84]. A decrease in drug clearance as well as a potential synergistic effect between GLP-1 receptor agonists and estrogens may contribute to enhanced central appetite suppression [85]. However, women are also more susceptible to gastrointestinal adverse events [5], which can lead to a higher rate of treatment discontinuation [86]. Nevertheless, evidence regarding sex-specific responses to pharmacological treatment remains limited, as many clinical trials do not report outcomes stratified by sex. To maximize efficacy, pharmacological interventions should be integrated with lifestyle modifications and tailored according to sex and obesity-associated complications to optimize long-term outcomes.

### Surgical Treatment

#### Overview of Metabolic-bariatric Surgery Techniques

In carefully selected candidates, bariatric surgery represents one of the most effective strategies for achieving sustained weight loss and improving obesity-associated complications [79]. Bariatric procedures are generally classified according to the anatomical and physiological modifications induced in the gastrointestinal tract, primarily involving restrictive and/or malabsorptive mechanisms.

The main surgical techniques include laparoscopic adjustable gastric banding, sleeve gastrectomy (SG), Roux-en-Y gastric bypass (RYGB) and biliopancreatic diversion with duodenal switch (BPD/DS). Laparoscopic adjustable gastric banding reduces gastric capacity but is associated with modest outcomes and a decline in use due to long-term complications [79]. SG is a restrictive procedure involving the resection of approximately 80% of the stomach, resulting in decreased ghrelin secretion, enhanced satiety, and improved glycemic control via increased GLP-1 release. RYGB combines restrictive and malabsorptive mechanisms by excluding the duodenum and proximal jejunum and induces favorable hormonal changes [79]. BPD/DS combines SG with an extensive intestinal bypass, yielding the greatest weight loss (70–90% excess weight loss) and highest rates of diabetes remission (> 90%), although this surgery is associated with increased risk of nutritional deficiencies and surgical complications [87]. This procedure is generally reserved for patients with severe obesity (BMI ≥ *5*0 kg/m²) [79].

#### Indications and Sex-Specific Considerations

Approximately 70–80% of bariatric procedures are performed in women [86]. Literature consistently attributes these disparities to gender-specific motivations, sociocultural stigma, and clinical referral patterns, rather than to intrinsic biological mechanisms. The predominance of women among metabolic-bariatric surgery candidates is therefore likely multifactorial. Potential contributing factors include sociocultural influences on body image and weight perception, greater healthcare-seeking behavior, and increased utilization of preventive medical care [88]. Women frequently exhibit body-image dissatisfaction, stigma associated with obesity, and psychosocial discomfort as primary reasons for surgical intervention, whereas men exhibit higher satisfaction and psychological well-being scores [89]. Additionally, women have a higher probability of being screened regarding metabolic-bariatric surgery since they are checked and counselled about weight-management techniques more regularly [90]. Biological factors, including sex-specific adipose tissue distribution, obesity-related quality-of-life impairment, and differential hormonal and metabolic responses, may also influence the need for metabolic-bariatric surgery. In addition, bariatric surgery positively affects female fertility, improving pregnancy and neonatal outcomes through weight loss and restoration of hormonal balance, particularly in women with polyendocrine metabolic ovarian syndrome (PMOS) [91, 92]. However, the relative contribution of sociocultural, healthcare-related, and biological mechanisms remains incomplete and warrants further investigation. Men undergoing bariatric surgery are typically older, have a higher preoperative BMI, and exhibit a greater prevalence of obesity-related complications, such as hypertension, hyperlipidemia, type 2 diabetes, and sleep apnea [93]. The primary motivation for patients undergoing bariatric surgery is the need to address current medical complications alongside preventive medical reasons [94]. Psychological and quality-of-life considerations, including improved self-esteem, enhanced mental health, and reductions in clothing size also feature prominently, though typically as secondary drivers [95].

Patients expectations associated with bariatric surgery often encompass substantial improvements in health and quality of life and may include unrealistic anticipations regarding weight loss [88]. Emerging evidence suggests that expectations and motivations differ by sex. Women tend to prioritize outcomes related to quality of life or physical appearance, whereas men emphasize survival-related motives, such as the improvement of obesity-associated complications or prevention of future health deterioration [96]. In a European multicenter study, women were more likely to expect that surgery alone would lead to relevant and sustained weight loss, whereas improvement of obesity-associated complications was a primary goal in both sexes [97]. Additionally, women reported higher preoperative anxiety and a greater desire for detailed information regarding postoperative lifestyle modifications and the risk of weight regain, highlighting the need for sex-specific preoperative counseling strategies [98].

Aligning patients expectations with realistic postoperative outcomes through tailored preoperative counseling is therefore crucial. Given the distinct motivational profiles and psychological needs observed between sexes, implementing sex-specific strategies during the preoperative assessment may enhance patient engagement, optimize adherence to postoperative recommendations, and ultimately improve long-term outcomes. 

Although current international guidelines do not recommend selecting bariatric procedures solely according to biological sex, increasing clinical evidence indicates that sex influences postoperative outcomes, including changes in body composition, metabolic improvement, nutritional requirements, and long-term weight maintenance. These observations suggest that biological sex should be considered as one of several factors informing individualized perioperative management, and postoperative follow-up. A better understanding of the biological mechanisms underlying these sex-specific responses may contribute to more personalized approaches to metabolic-bariatric surgery [99]. 

## Sex Differences in Outcomes after Bariatric Surgery

### Sex-Specific Trajectories of Weight Loss after Bariatric Surgery

Sex-specific differences in weight loss trajectories after bariatric surgery have been reported, although findings remain heterogeneous [100]. Several studies suggest that men may achieve greater absolute weight loss and a higher percentage of excess weight loss (%EWL) compared to women [95]. In line with these findings, a higher absolute weight loss in men has been reported, although differences in relative %EWL did not reach statistical significance, likely due to the reduced sample size [101].

Conversely, other studies have reported greater relative weight loss in women, particularly after RYGB [99]. Over long-term follow-up (5 years), weight regain was similar between RYGB and SG in both sexes, although men showed less regain following SG. Notably, women undergoing RYGB consistently achieved greater weight loss than with SG at all-time points, whereas in men, outcomes for both techniques tended to converge earlier. In contrast, Popa et al. [102] identified a tendency for a greater weight loss in men than women, although differences did not reach statistical significance. Other studies have reported heterogeneous findings. Some have observed greater overall weight loss in women. Similarly, higher %EWL among women following bariatric surgery has been found, although this difference disappeared after propensity score matching, indicating that, once baseline BMI is accounted for, weight-loss outcomes were comparable between sexes [89]. Only few studies stratify women by menopausal status, despite the possibility that premenopausal and postmenopausal women differ in baseline visceral adiposity, skeletal muscle reserve, resting energy expenditure, and inflammatory burden. This omission may partly explain inconsistent findings regarding whether women or men achieve greater relative weight loss after surgery.

**Fig. 2:**
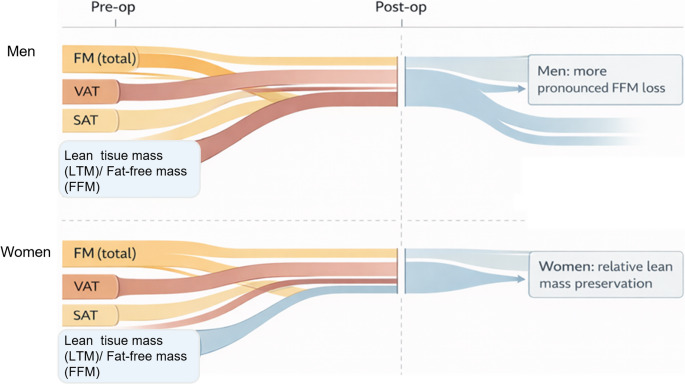
Visual representation of body composition modifications using a Sankey diagram. Proportional transitions between categories are represented, displaying the relative distribution of fat mass, visceral adipose tissue, subcutaneous adipose tissue, and lean/fat-free mass from baseline to the post-operative period. Men (upper panel) exhibit a disproportionate loss of fat free mass relative to total weight loss, whereas women (lower panel) demonstrate greater relative preservation of lean mass, indicating sex-specific metabolic responses to bariatric surgery. Abbreviations: FM, fat mass; FFM, fat-free mass; LTM, lean tissue mass; SAT, subcutaneous adipose tissue; VAT, visceral adipose tissue

Taken together, these findings illustrate the heterogeneity in weight-loss outcomes when stratified by sex, limiting definitive conclusions. While short-term analyses often suggest an advantage for women in terms of %EWL, certain techniques, such as SG, may favor men. However, long term trajectories tend to converge between sexes, highlighting the relevance of individualized surgical approaches (Fig. 2).

### Sex-Specific Body Composition Changes after Bariatric Surgery

**Fig. 3:**
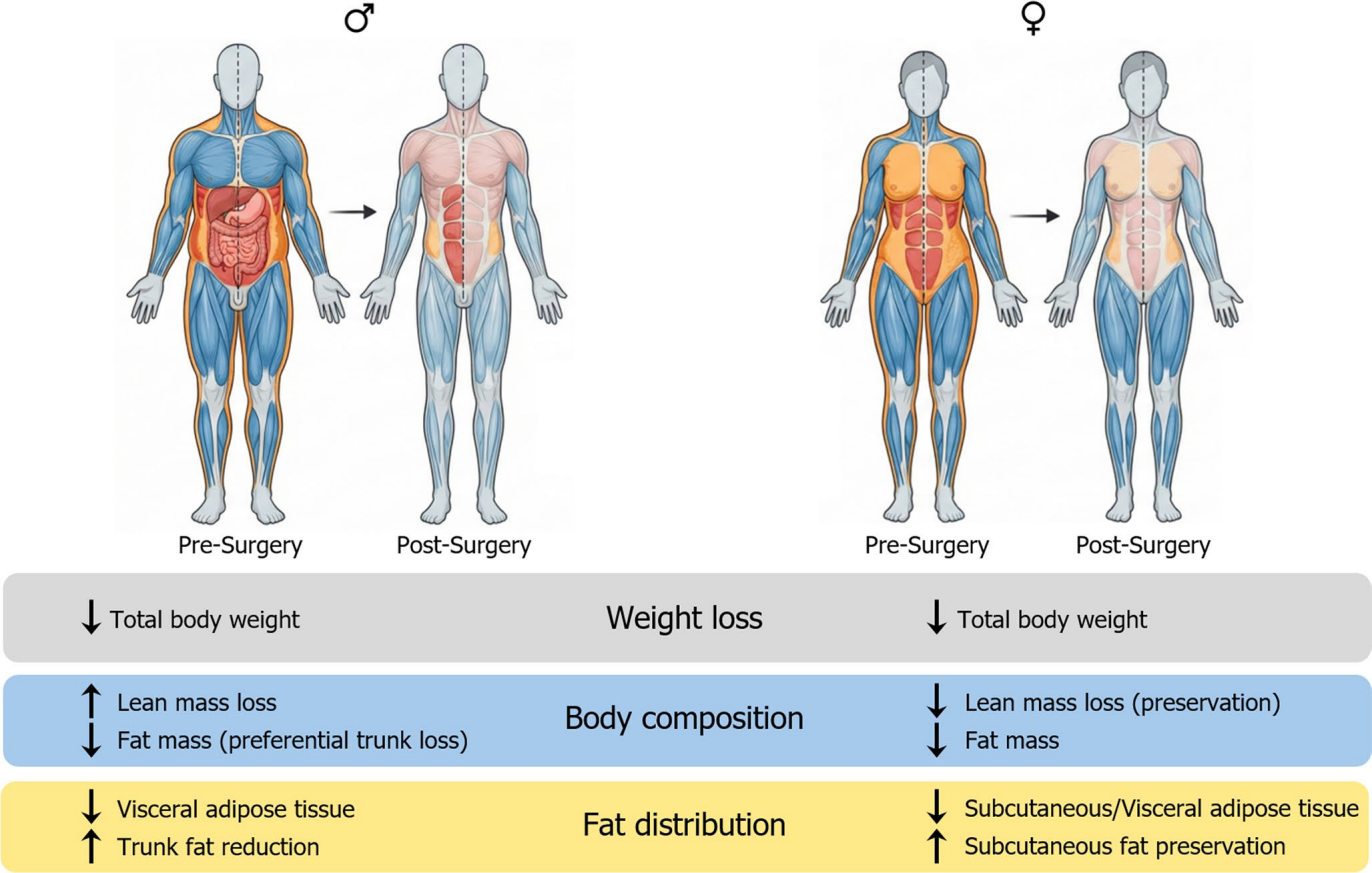
Comparative impact of bariatric surgery on body composition and fat distribution between men and women. While both sexes achieve significant reductions in total body weight and excess weight loss percentage, sexual dimorphism is evident in tissue-specific changes. Men exhibit a more pronounced loss of lean mass and a preferential reduction in trunk/visceral fat. Conversely, women demonstrate relative preservation of lean mass and subcutaneous fat depots. These differences highlight the sex-specific nature of fat redistribution and the maintenance of metabolically active lean tissue following surgical intervention. EWL, excess weight loss

Bariatric surgery induces significant modifications in body composition, characterized by reductions in total body weight, fat mass (FM), and visceral adipose tissue, while often preserving or relatively increasing lean tissue mass (LTM) [103, 104]. These compositional changes contribute substantially to metabolic improvements, including enhanced insulin sensitivity, glycemic control, lipid profile, and blood pressure regulation [105], although the magnitude and distribution of these changes differ between sexes. Before surgery, men typically present higher absolute amounts of both LTM and FM compared with women. Following bariatric surgery, men tend to experience a greater reduction in total and regional fat mass particularly in the trunk and upper extremities and exhibit a higher post-surgical LTM/FM ratio in the trunk region [101]. Although the overall percentage of total weight loss does not differ significantly between sexes, men show a more pronounced reduction in fat-free mass than women [106]. These sex-specific patterns of body composition changes may have relevant implications for postoperative metabolic outcomes and functional status. The preferential loss of trunk fat and higher LTM/FM ratio in men may contribute to greater improvements in insulin sensitivity and cardiometabolic risk, whereas the relative preservation of lean mass in women may support physical function and energy expenditure. Collectively, these observations reveal the relevance of considering sex as a biological variable in evaluating bariatric surgery outcomes and highlight the potential for tailored perioperative strategies to optimize body composition and metabolic benefits in both men and women. Pre- to post-operative transitions in body composition compartments are shown in Fig. 3.

Sex hormones appear to modulate these outcomes. Importantly, menopausal status likely represents a major modifier of these responses, given the profound effects of estrogen decline on adipose tissue distribution, inflammation, and skeletal muscle preservation. Estrogen signaling through muscle-specific ERα and estrogen-related receptors is thought to preserve muscle mass and quality after bariatric surgery by upregulating genes involved in fatty acid metabolism and insulin sensitivity [107]. In postmenopausal women, hormone replacement therapy has been shown to attenuate or reverse sarcopenia and fat accumulation, improving muscle quality and function [44, 108, 109]. Notably, the beneficial effects of hormone replacement therapy may persist even after treatment discontinuation [110].

Taken together, these findings highlight that sex-specific hormonal and body composition characteristics critically influence the metabolic and structural outcomes of bariatric surgery. Recognizing and accounting for these differences is essential for interpreting post-surgical responses, optimizing individualized treatment strategies, and improving long-term outcomes for both men and women. These sex-specific differences in body composition also impact on adipokine secretion, further contributing to differential adaptations after bariatric interventions.

### Adipokine Responses and Inflammation

Beyond changes in body weight and composition, bariatric surgery also induces changes in the endocrine function of adipose tissue. Reported modifications in adipokine secretion include reductions in leptin and other inflammatory markers such as IL-6 and TNF-α, as well as increases in adiponectin and gut-derived peptides, including GLP-1 and PYY [111, 112]. Although these changes are observed in both sexes, their magnitude and physiological implications may differ. To contextualize the available evidence, Table 1 provides a comprehensive overview of adipokine biological functions, reported sex-specific differences, obesity-associated alterations, and the effects of bariatric surgery on their circulating concentrations.

**Table 1:** Summary of characteristics of some selected adipokines

Adipokine	Principal metabolic role	Sex/menopause considerations	Obesity-associated pattern	Postoperative pattern after metabolic-bariatric surgery	Limitations	References
Leptin	· Signals energy stores to central nervous system. · Regulates appetite, energy expenditure, neuroendocrine function, and lipolysis.	· Women: Higher (↑ subcutaneous fat). · Menopause: Alters levels via visceral adiposity and leptin sensitivit	· Hyperleptinemia with central/peripheral leptin resistance. · Levels correlate positively with adiposity.	· Marked decline, proportional to fat mass los	· Interpret relative to fat mass, distribution, and sensitivity, not concentration alone.	[113, 145, 146]
Adiponectin	· Insulin-sensitizing, anti-inflammatory, and anti-atherogenic. · Involved in glucose and lipid metabolism.	· Women: Typically higher. · Menopause: Visceral adiposity and inflammation may attenuate favorable profile.	· Decreased. · Inversely associated with insulin resistance, visceral adiposity, and cardiometabolic.risk.	· Increases. · Parallels improved insulin sensitivity and reduced inflammatory burden.	· Sex postoperative differences poorly characterized. · HMW adiponectin may be more informative than total.	[147, 148]
Ghrelin	· Orexigenic hormone · Regulates hunger, meal initiation, growth hormone secretion, and energy balance	· Women: Higher preprandial levels and faster postprandial suppression. · Menopause: Effects remain poorly defined.	· Decreased (fasting levels). · Impaired meal-related dynamics.	· ↓ after SG. · Variable after RYGB (depends on anatomy, vagal signaling, and follow-up duration).	· Strongly procedure-dependent. · Total and acylated ghrelin should be distinguished.	[147, 149]
Visfatin / NAMPT	· Involved in NAD⁺ biosynthesis, metabolism, inflammation, and glucose homeostasis.	· No consistent circulating sexual dimorphism. · Depot-specific expression may be more relevant than sex.	· Variable. · Associated with visceral adiposity, inflammation, and insulin resistance.	· Heterogeneous. · Not clearly procedure-specific	· Circulating levels may not reflect depot-specific expression or intracellular NAMPT activity.	[145, 150]
Resistin	· Pro-inflammatory mediator linked to macrophage activation, insulin resistance, and vascular risk.	· No consistent sex differences. · Menopause inflammatory remodeling may be relevant but understudied.	· Variable / Elevated. · Often ↑ in obesity and metabolic dysfunction.	· Inconsistent. · May decrease after substantial weight loss.	· Reflects immune-cell activity. · Interpretation must consider inflammatory status and assay variability.	[146, 148]
Growth hormone (GH)	· Regulates lipolysis, fat oxidation, protein turnover, muscle maintenance, and glucose metabolism	· Sexually dimorphic (influenced by estrogen). · Menopause: May affect GH-related lean mass and metabolic responses	· Functional hyposomatotropism. · Reduced GH secretion and altered pulsatility.	· Partial restoration of GH secretion and GH/IGF-1 axis after substantial weight loss	·Age, adiposity, insulin resistance, menopausal status, and lean mass loss confound interpretation.	[66, 151]

#### Leptin

Baseline leptin levels are higher in women than in men due to their greater adiposity, although the interpretation of leptin concentrations in women should account for menopausal status given that estrogen decline and visceral adiposity redistribution may alter the relationship between fat mass, leptin secretion, and leptin sensitivity [113]. Leptin levels decrease substantially after bariatric surgery, in line with reductions in adipose tissue mass [112]. Supporting these observations, rodent models of diet-induced obesity also show postoperative reductions in circulating leptin after bariatric procedures in both males and females [114, 115]. However, most of the preclinical studies have been conducted regarding one sex only. Consequently, potential sex-specific differences in the magnitude or temporal profile of the leptin response remain to be fully elucidated.

#### Adiponectin

Adiponectin levels progressively increase after bariatric surgery, accompanied by improvements in insulin sensitivity, reduced inflammation, and enhanced metabolic control [112]. In preclinical models, females exhibit higher baseline adiponectin concentrations, and a more pronounced postoperative increase when matched for equivalent weight loss [116]. Evidence of this sexual dimorphism in humans is still limited and requires further investigation. The adiponectin-to-leptin ratio also improves after bariatric surgery, serving as a marker of enhanced body composition and metabolic status [117]. Although growing evidence supports sex-related differences in adipokine regulation after bariatric surgery, current evidence remains limited.

#### Ghrelin

The change in ghrelin levels following bariatric surgery remains a matter of debate. Variability in surgical techniques, including the extent of vagal denervation, the length of the alimentary limb, the size of the gastric pouch as well as differences in weight loss contribute to marked heterogeneity across studies and to conflicting results [118]. Taking this heterogeneity into account, a meta-analysis of 325 patients reported a decrease in ghrelin levels during the first three months after surgery, followed by a gradual increase beyond this period [119]. Regarding sex, no consistent differences have been reported.

#### Growth Hormone

Bariatric surgery represents a potent stimulus for the restoration of the GH/IGF-1 axis, effectively reversing obesity-associated functional hyposomatotropism. The postoperative recovery of GH secretion is critical for optimizing fat oxidation and preserving lean mass during periods of rapid weight loss [120]. Comparative studies indicate that, although GH pulsatility is restored in both sexes, the magnitude of the increase in insulin-like growth factor 1 relative to weight loss may differ, with some evidence suggesting an earlier response in women potentially due to their higher baseline estrogenic tone [121].

Other adipokines, including resistin and visfatin, have not received as much attention as those described above. Findings for these adipokines are highly heterogeneous, and meta-analyses have generally failed to demonstrate robust postoperative changes [122]. Noteworthy, the participation of other factors cannot be ruled out, as is the case for membrane proteins and inflammatory-related molecules [123–125]. Regarding sexual dimorphism, most analyzed cohorts are predominantly single-sex, and when both sexes are included, sex-stratified investigation is generally lacking. Further studies are, therefore, required to elucidate the role of these adipokines in post-bariatric metabolic adaptations and to determine whether sex-specific differences contribute to long-term outcomes. Importantly, the marked heterogeneity among studies may reflect differences in surgical procedures, postoperative follow-up duration, menopausal status, baseline adipose tissue distribution, and the underrepresentation of men in most bariatric cohorts. Stratification by menopausal status is particularly relevant since the menopause transition modifies adipose tissue distribution, inflammatory burden, insulin sensitivity, and endocrine regulation, all of which influence all adipokines.

Altogether, these factors complicate direct comparisons and might partly explain the inconsistent magnitude of postoperative adipokine responses reported across studies.

### Functional Remodeling of Adipose Tissue

**Fig. 4:**
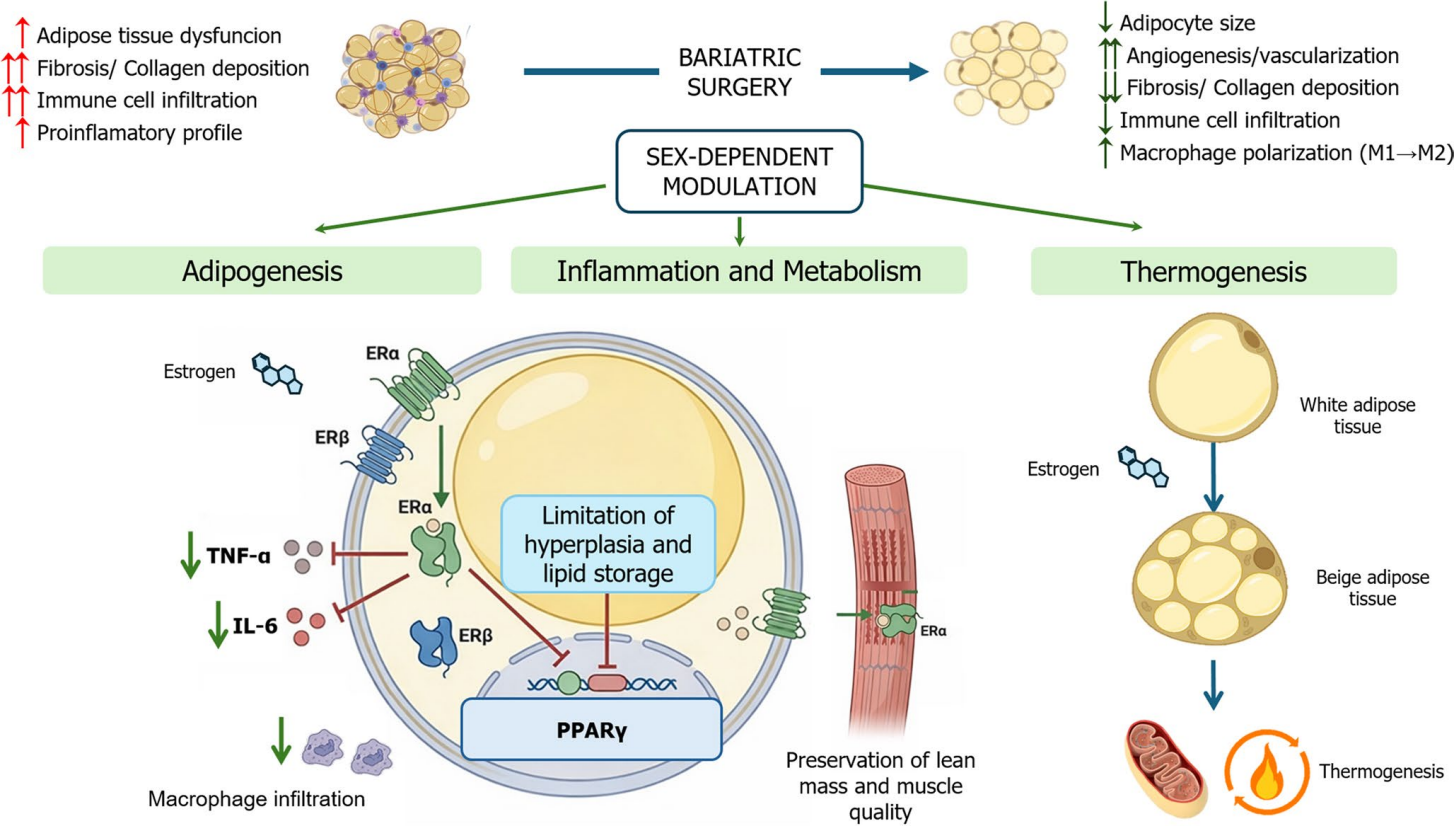
Metabolic-bariatric surgery mitigates obesity-associated adipose tissue dysfunction by reducing adipocyte hypertrophy, fibrosis and collagen deposition, and immune cell infiltration, accompanied by a shift in macrophage polarization from a pro-inflammatory (M1) to an anti-inflammatory (M2) phenotype and improved tissue vascularization. Sex-specific modulation of these responses is mediated, in part, by estrogen signalling via estrogen receptor-α (ERα) and estrogen receptor-β (ERβ), which regulate lipid storage capacity and restrain adipogenesis through inhibition of key adipogenic regulators, including PPARγ, thereby shaping depot-specific fat distribution. In addition, estrogen action suppresses the production of pro-inflammatory cytokines, such as TNF-α and IL-6, and promotes thermogenic remodelling through white-to-beige adipose tissue conversion, collectively supporting improved metabolic homeostasis following surgery. Abbreviations: TNF-α, Tumor Necrosis Factor alpha; IL-6, Interleukin 6; PPARγ, Peroxisome Proliferator-Activated Receptor γ

Beyond fat mass reduction, bariatric surgery induces cellular and structural changes in adipose tissue, including alterations in adipocyte size and turnover, angiogenesis, fibrosis, mechanical properties and immune cell infiltration [126–129]. Bariatric surgery triggers a profound qualitative remodeling of adipose tissue, a process modulated by sex-specific hormonal signaling as depicted in Fig. 4. A key mechanism involves a shift in macrophage polarization, from the pro-inflammatory M1 phenotype to the anti-inflammatory M2 phenotype [128]. Although sex-stratified studies in humans remain limited, evidence suggests that women experience a greater reduction in inflammation-related parameters, such as C-reactive protein, following bariatric surgery [130]. Additionally, estrogens promote a M2 macrophage differentiation within the subcutaneous adipose tissue in women after weight loss [131]. Altogether, these findings indicate a modulatory role of sex hormones, although postoperative cohorts explicitly comparing women and men are still needed. However, interpretation of these findings remains challenging due to important inter-study variability in adipose tissue depot analysis, inflammatory marker assessment, hormonal status, and postoperative evaluation timepoints. In particular, differences in menopausal status and visceral-to-subcutaneous adipose tissue distribution may substantially influence postoperative inflammatory remodeling as well as thermogenic adaptations, contributing to the inconsistent sex-related effects reported across studies.

Estrogens also modulate key aspects of this remodeling process, such as vascularization and inflammation [34, 132]. Estrogens enhance angiogenesis by modulating factors such as vascular endothelial growth factor (VEGF), endothelial nitric oxide synthase (eNOS), and Akt via ERα and ERβ receptors expressed in the vascular endothelium [133]. Furthermore, estrogens exert antifibrotic effects by limiting collagen deposition. Consistent with this notion, *Era* knockout murine models exhibit a marked increase in fibrosis in both sexes compared with wild-type counterparts [134].

Beyond the structural and inflammatory adaptations described above, bariatric surgery also remodels the thermogenic function of adipose tissue. The increase in total energy expenditure and thermogenesis observed after bariatric surgery appears to be primarily mediated by the beiging of white adipose tissue rather than by the expansion of classical brown adipose tissue depots [135, 136]. Multiple rodent studies subjected to RYGB and SG show induction of a thermogenic gene profile in white adipose depots, characterized by increased expression of *Ucp1*, peroxisome proliferator-activated receptor gamma coactivator 1-alpha (*Pgc1α*) *or* PR domain containing 16 (*Prdm16*), leading to enhanced mitochondrial activity in both subcutaneous and visceral adipose tissue [137–139]. In addition, an increase in brown adipose tissue activity through thermogenesis and energy expenditure mediated by PGC-1α has been observed in studies conducted in rats undergoing one-anastomosis gastric bypass [140, 141]. These findings suggest that the metabolic benefits of bariatric surgery extend beyond weight loss and involve a qualitative remodeling of adipose tissue toward a more metabolically active phenotype.

Menopausal status may be particularly relevant to these thermogenic adaptations. Experimental and human evidence indicates that estrogenic signaling contributes to brown adipose tissue activity and white adipose tissue beiging, partly through ERα-dependent and sympathetic mechanisms [73].

Overall, current evidence indicates that bariatric surgery induces a complex remodeling of adipose thermogenic function, characterized by preservation of brown adipose tissue and enhanced white adipose beiging. Nonetheless, data remain inconclusive, particularly in humans, highlighting the need for longitudinal, sex-stratified studies integrating molecular, imaging, and metabolic endpoints to elucidate the precise role of thermogenic mechanisms in post-surgical metabolic improvement. The variability among available studies may be partially explained by differences in species, adipose tissue depot analyzed, environmental temperature conditions, surgical technique, and duration of postoperative follow-up. Moreover, the influence of sex hormones and menopausal status on thermogenic activation remains insufficiently characterized in human cohorts.

### Sex Differences in Obesity-Associated Complications after Bariatric Surgery

Metabolic-bariatric surgery leads to high remission rates of obesity-associated complications [142]. As previously noted, however, men usually undergo bariatric surgery at an older age and with a greater burden of complications than women, who, as previously mentioned, represent the majority of bariatric surgery candidates. These baseline differences should be considered when interpreting postoperative outcomes. Regarding type 2 diabetes, sex-matched analysis indicates comparable remission rates between men and women. Likewise, no significant sex-related differences have been reported in the remission of arterial hypertension or dyslipidemia, nor in the resolution of obstructive sleep apnea. In contrast, remission of metabolic syndrome seems to be more frequent in men than in women, probably driven by their greater reduction in visceral adipose tissue [143, 144].

Taken together, current evidence suggests that once obesity-related complications are established, biological sex exerts a modest influence on the probability of remission, reinforcing the notion that sex differences in postoperative outcomes may be due to disparities in baseline clinical profiles rather than intrinsic differences in surgical responsiveness. Importantly, evaluating postoperative metabolic responses should consider that men and women frequently differ in age, adiposity distribution, obesity-associated complications, and procedure allocation. These factors may confound the attribution of metabolic responses to biological sex and likely contribute to discrepancies observed across clinical studies.

## Interpretation Pitfalls and Sources of Bias

The interpretation of sex-related differences following bariatric surgery is complex, and multiple sources of bias must be considered. First, important baseline disparities exist between men and women, with men typically undergoing surgery at an older age, with higher preoperative BMI and increased obesity-related complications. These baseline differences may influence not only postoperative trajectories but also confound the attribution of outcomes to biological sex alone.

Furthermore, sexual dimorphism in adipose tissue distribution represents an additional source of confounding, since men typically exhibit a predominance of visceral adipose tissue, strongly associated with a pro-inflammatory state and increased insulin resistance. In contrast, premenopausal women predominantly accumulate subcutaneous adipose tissue, a depot generally linked to a more favorable inflammatory and metabolic profile. Consequently, postoperative metabolic improvements may partly reflect depot-specific fat loss rather than intrinsic sex-related differences in surgical responsiveness.

Lack of stratification by menopausal status represents a particularly important source of misclassification within female cohorts, since ovarian hormones modify visceral adiposity, adipose tissue inflammation, adipokine secretion, skeletal muscle maintenance, and thermogenic activity. Consequently, analyses that compare “men” versus “women” without distinguishing premenopausal and postmenopausal women may obscure biologically meaningful differences and contribute to inconsistent findings across studies.

Regarding surgical procedures, an unequal sex distribution across the two most commonly performed techniques, SG and RYGB, exists. Differences in procedure allocation may confound sex comparisons, potentially exaggerating or attenuating apparent sex-specific effects. Furthermore, variability in reporting weight loss or diabetes remission criteria may reflect methodological inconsistency rather than true biological dimorphism. Additionally, men remain underrepresented in bariatric cohorts, limiting statistical power for robust sex-stratified analyses.

Taken together, these sources of bias suggest that reported sex disparities should be interpreted within the context of baseline phenotype, hormonal milieu, and study design, emphasizing the need for standardized and sex-stratified analyses.

## Clinical Implications and Future Directions

Sex-related differences should be considered in the clinical decision-making process for bariatric surgery, from preoperative evaluation to postoperative follow-up. Despite current international guidelines not recommending these lection bariatric procedures according to biological sex alone, accumulating evidence in the clinical setting indicates that sex influences postoperative metabolic adaptation, body composition changes, and long-term outcomes [99]. Accordingly, incorporating sex as a biological variable may improve risk stratification, patient counseling, andindividualized perioperative management. Incorporating sex as a biological variable may improve procedure selection and enhance outcome prediction. Future research should prioritize adequately powered, sex-stratified studies to elucidate the underlying mechanisms thereby, supporting the development of personalized, evidence-based strategies for obesity management.

Collectively, bariatric surgery induces coordinated changes in adipokines and inflammatory mediators that support metabolic improvement. Sex-specific differences in baseline adipokine profiles, hormone interactions, and adipose tissue distribution influence both the magnitude and temporal profile of these responses. Women, with higher baseline leptin and adiponectin levels, may experience more pronounced relative changes, while men’s reductions in visceral fat and associated inflammatory mediators may confer distinct cardiometabolic benefits. These observations underscore the need to consider sex as a biological variable in evaluating post-bariatric metabolic adaptations and for tailoring perioperative strategies to optimize endocrine and inflammatory recovery.

From a clinical perspective, recognition of sex-specific metabolic adaptations may contribute to more individualized perioperative management strategies. Differences in body composition, adipokine responses, and inflammatory remodeling could influence nutritional follow-up, preservation of lean mass and postoperative monitoring of metabolic complications. In women, menopausal status should beconsidered when interpreting postoperative metabolic responses, as estrogen deficiency may influence body composition,insulin sensitivity, and adipose tissue function after surgery (153). Preservation of lean body mass has emerged as animportant determinant of long-term functional outcomes, supporting individualized nutritional interventions and structuredexercise programs during postoperative follow-up (154). Collectively, these observations reinforce the concept that longtermmanagement after bariatric surgery should extend beyond weight loss alone and incorporate biological sex,hormonal status, and body composition to optimize patient-centred outcomes. In women, menopausal status may deserve particular consideration when interpreting postoperative metabolic responses and planning long-term follow-up. Furthermore, the underrepresentation of men in bariatric cohorts highlights the need to improve sex-balanced recruitment in future clinical studies.

## Conclusion

Metabolic-bariatric surgery induces profound and sustained metabolic benefits in people with obesity; however, growing evidence highlights that biological sex modulates several dimensions of the postoperative response, including adipose tissue remodeling, adipokine dynamics, inflammatory adaptations, and changes in body composition. Importantly, the direction and magnitude of reported sex-related differences are not entirely consistent across studies, reflecting the influence of baseline disparities in age, adiposity distribution, and burden of complications. In women, menopausal status likely contributes to variability in adipose tissue remodeling, inflammatory adaptations, and thermogenic responses. Moreover, differences in surgical procedures and duration of follow-up complicate direct sex comparisons. Collectively, the available evidence supports the concept that biological sex represents a relevant modifier of metabolic adaptation after bariatric surgery, although these effects must be interpreted with caution given the substantial clinical and methodological heterogeneity across available studies.

## Data Availability

No datasets were generated or analysed during the current study.

## Abbreviations

AT: adipose tissue
BPD/DS: biliopancreatic diversion with duodenal switch
BMI: body mass index
eNOS: endothelial nitric oxide synthase
ERα: estrogen receptor alpha
ERβ: estrogen receptor beta
%EWL: excess weight loss
FM: fat mass
IL-6: interleukin-6
GLP-1: glucagon-Like Peptide-1
LTM: lean tissue mass
MeSH: Medical Subject Headings
NAD: nicotinamide adenine dinucleotide
RYGB: roux-en-Y gastric bypass
SG: sleeve gastrectomy
TBX1: T-box transcription factor 1
TNF-α: tumor necrosis factor-alpha
TMEM26: transmembrane protein 26
UCP1: uncoupling protein 1
VEGF: vascular endothelial growth factor
WAT: white adipose tissue
